# Supplementary material for: MetaboLabPy—An Open-Source Software Package for Metabolomics NMR Data Processing and Metabolic Tracer Data Analysis
Source: Metabolites. 2025 Jan 14;15(1):48. doi: 10.3390/metabo15010048 (PMC11767891; doi:10.3390/metabo15010048)
Supplement: Supplementary file 1 [file metabolites-15-00048-s001.zip › metabolites-3401492-supplementary.pdf]

Supplementary Materials

MetaboLabPy – an open-source software package for metabolomics NMR-data processing and metabolic tracer data analysis

Christian Ludwig [1,*]

[1] Department of Metabolism and Systems Sciences School of Medical Sciences, College of Medicine and Health, University of Birmingham, United Kingdom; C.Ludwig@bham.ac.uk

* Correspondence: C.Ludwig@bham.ac.uk

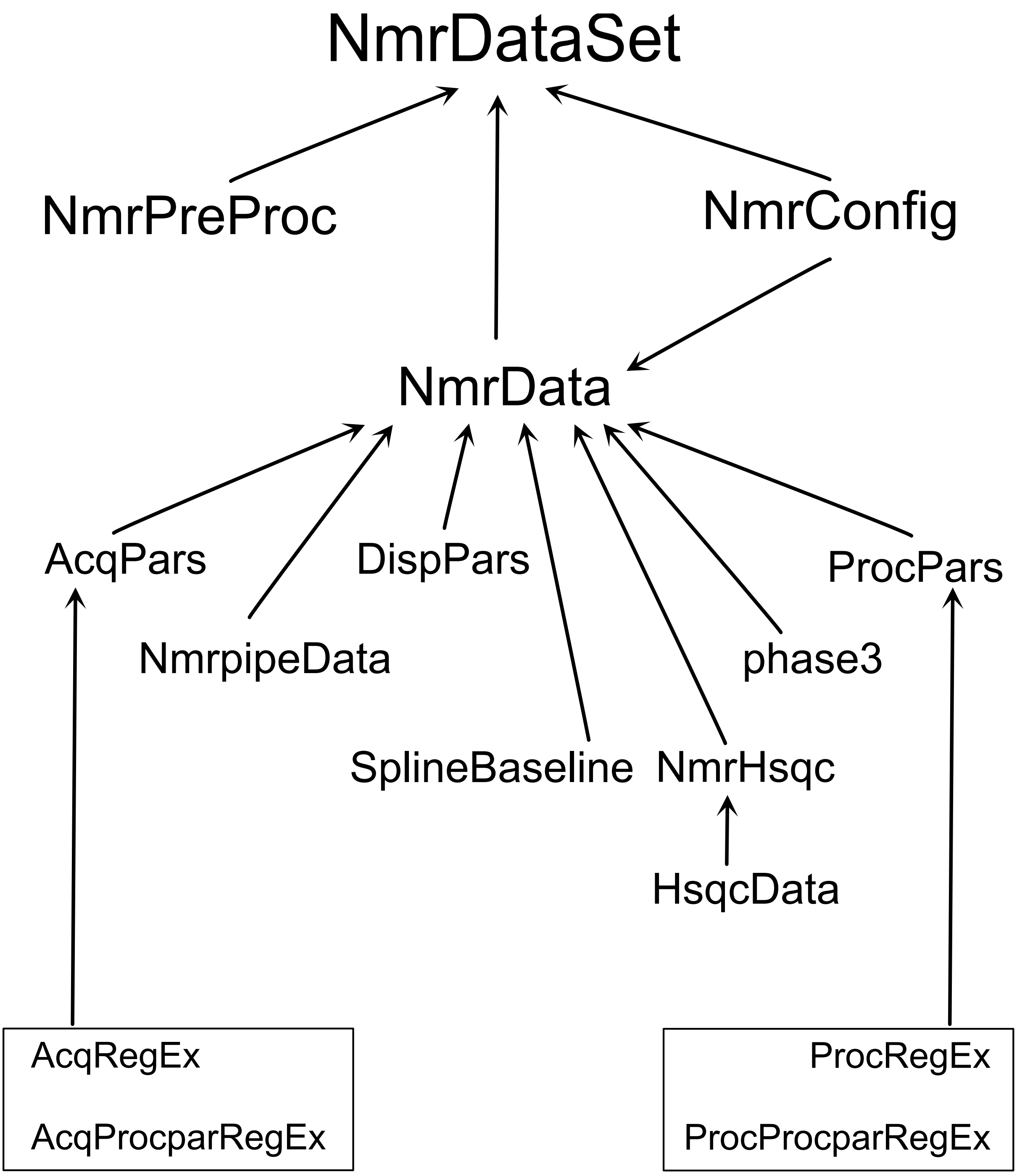

Figure S1. schematic plot of the class structure of MetaboLabPy.

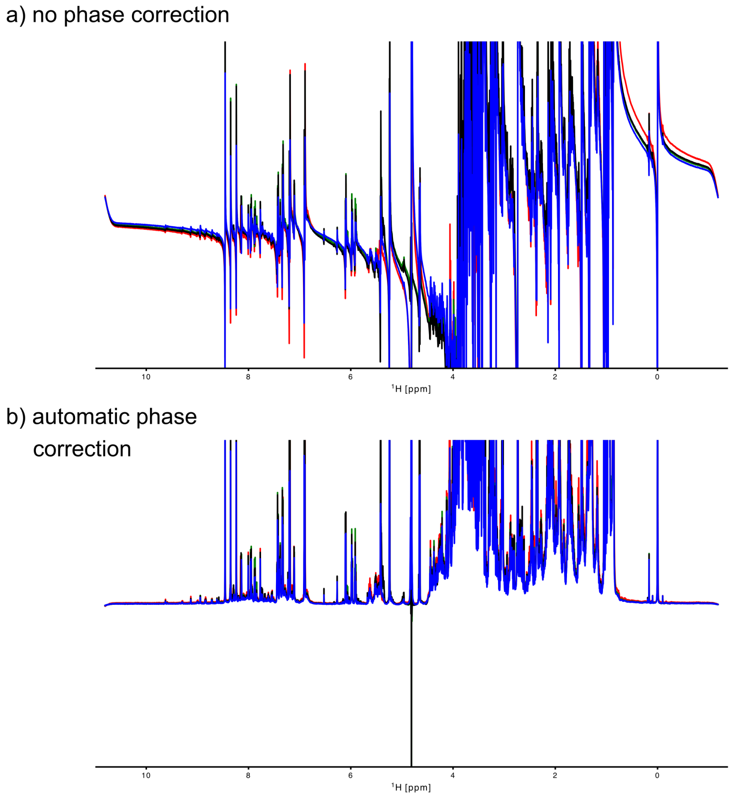

Figure S2. Automatic phase correction of *Daphnia* extract NMR spectra - 1. The spectra were acquired on a 600 MHz Bruker Avance III system equipped with a 1.7 mm CryoProbe probe. Panel a) shows the NMR spectra before phase correction. Panel b) depicts the spectra after automatic phase correction using the algorithm described in [1].

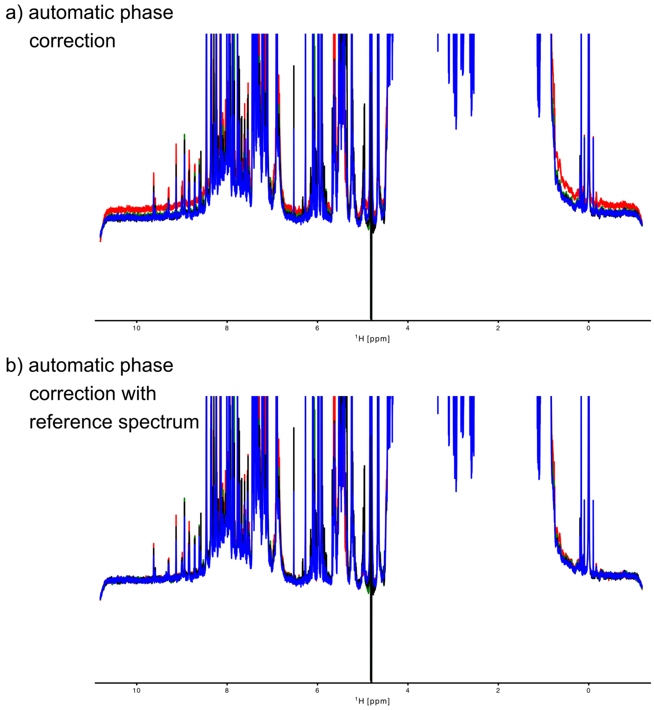

Figure S3. Automatic phase correction of *Daphnia* extract NMR spectra - 2. The spectra were acquired on a 600 MHz Bruker Avance III system equipped with a 1.7 mm CryoProbe probe. Panel a) shows an expanded view of Figure S1 panel b). Panel b) depicts the spectra after automatic phase correction using the manually phase corrected blue spectrum as reference data, minimizing baseline regions at both edges of the spectra.

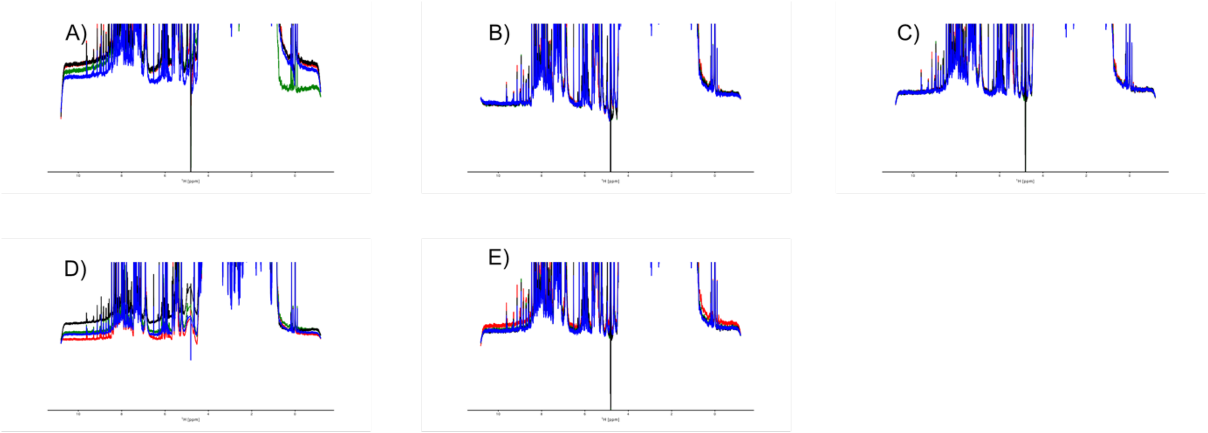

Figure S4. Automatic phase correction of *Daphnia* extract NMR spectra. The spectra were acquired on a 600 MHz Bruker Avance III system equipped with a 1.7 mm CryoProbe probe. Panel A) spectra were automatically phase corrected using the MNova software. Panel B) TopSpin (apk0.noe), panel C) MetaboLabPy using reference spectrum and baseline-based algorithm. Panel D) MetaboLab's apk5 algorithm. Panel E) MetaboLabPy's automatic phase correction algorithm, described in [1]

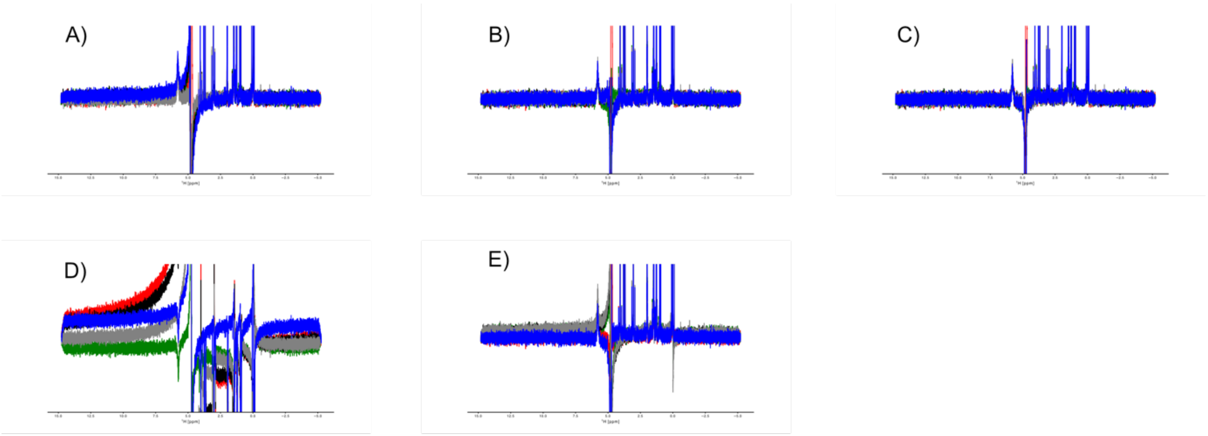

Figure S5. Automatic phase correction of a metabolite mix NMR spectra. The spectra were acquired on a 600 MHz Bruker IVDr equipped with a room-temperature inverse probe. Panel A) spectra were automatically phase corrected using the MNova software. Panel B) TopSpin (apk0.noe). Panel C) MetaboLabPy using reference spectrum and baseline-based algorithm. Panel D) MetaboLab's apk5 algorithm. Panel E) MetaboLabPy's automatic phase correction algorithm, described in [1]

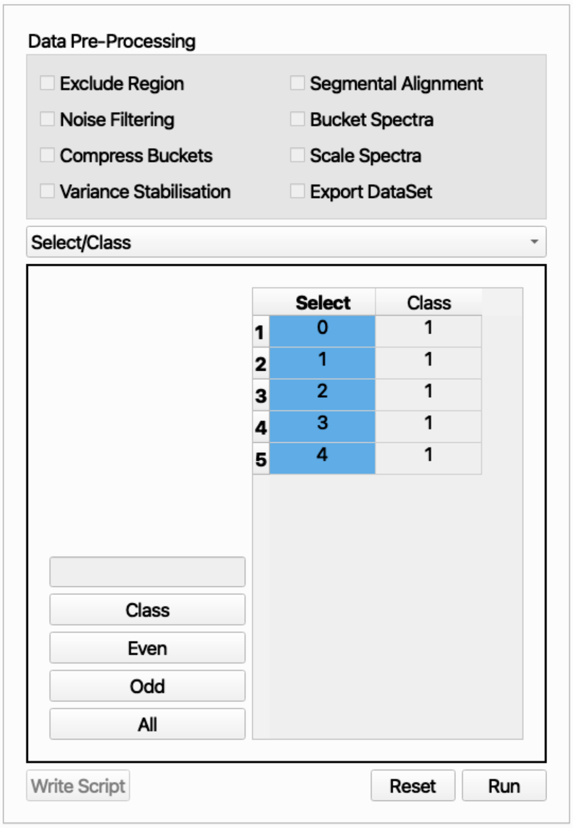

Figure S6. Graphical user interface element for data pre-processing. The user can select the different sections. Whenever areas have to be selected in the spectra, the user can do this clicking with the mouse.

	A	B	C	D	E	F	G	H	I	J	K	L	M	N	O	P	Q	R
1	Dataset.0	Experiment	Multiplet.0	Percentage	Intensity.0	HSQC.0	Dataset.1	Experiment	Multiplet.1	Percentage	Intensity.1	HSQC.1	Dataset.2	Experiment	Multiplet.2	Percentage	Intensity.2	HSQC.2
2	1	1	2	4.556	22654979.4	0 1 1	1	2	2	5.1009	147098.94	0 1 1	1	3	2	4.843	21730166.4	0 1 1
3			2, 1	0.846		n_bonds: 1			2, 1	0.0		n_bonds: 1			2, 1	0.0		n_bonds: 1
4			2, 3	94.598					2, 3	94.8991					2, 3	95.157		
5			2, 1, 3	0.0					2, 1, 3	0.0					2, 1, 3	0.0		
6			3	14.348	120420185.24921805				3	15.509	43498041.31897458				3	14.233	162424759.24583393	
7			3, 2	85.652					3, 2	84.491					3, 2	85.767		

Figure S7. Exported multiplet data as input for isotopomer analysis. Columns A & B show dataset and experiment of the NMR experiment. Column C describes which multiplet components are analysed. Column D shows the corresponding percentages. Column E contains the peak intensities for each multiplet. Column F shows which carbons contribute to a normal HSQC spectrum and how many bonds were considered to contribute to the multiplet when fitting the multiplet. This pattern is then repeated for further NMR spectra.

	A	B	C	D	E	F	G	H	I	J	K	L
1	Dataset.0	Experiment	Isotopologu	Percentage	Dataset.1	Experiment	Isotopologu	Percentage	Dataset.2	Experiment	Isotopologu	Percentages.2
2	1	1	0	73.17	1	2	0	73.17	1	3	0	73.17
3			1	4.01			1	4.01			1	4.01
4			2	22.52			2	22.52			2	22.52
5			3	0.3			3	0.3			3	0.3

Figure S8. GC-MS data as input file for isotopomer analysis. Columns A & B show dataset and experiment number of the corresponding NMR dataset. Column C plots the mass isotopologue (0 for m+0, 1 for m+1 etc). Columns E-H contain the same data for the second experiment etc.

	A	B	C	D	E	F	G	H	I	J
1	Experiment	Fitted Isoto	Fitted Isoto	Multiplets	Exp. %	Sim. %	Exp. GC-MS	Sim. GC-MS	Exp. NMR1I	Sim. NMR1D %
2	1	[0, 0, 0]	73.95	[2]	5.05	3.34	[73.17 4.0	[73.95 3.16 22.9 0.]		
3		[0, 0, 1]	3.16	[2, 1]	0.71	0.00				
4		[0, 1, 1]	22.90	[2, 3]	93.67	95.63				
5				[2, 1, 3]	0.57	1.03				
6				[3]	14.35	14.58				
7				[3, 2]	85.65	85.42				
8	2	[0, 0, 0]	73.88	[2]	5.26	3.36	[73.17 4.0	[73.88 3.45 22.67 0.]		
9		[0, 0, 1]	3.45	[2, 1]	0.59	0.00				
10		[0, 1, 1]	22.67	[2, 3]	93.36	95.60				
11				[2, 1, 3]	0.79	1.03				
12				[3]	15.46	15.63				
13				[3, 2]	84.54	84.37				
14	3	[0, 0, 0]	73.67	[2]	4.07	3.29	[73.17 4.0	[73.67 3.16 23.17 0.]		
15		[0, 0, 1]	3.16	[2, 1]	0.00	0.00				
16		[0, 1, 1]	23.17	[2, 3]	95.93	95.68				
17				[2, 1, 3]	0.00	1.03				
18				[3]	14.23	14.45				
19				[3, 2]	85.77	85.55				
20										

Figure S9. Exported isotopomer data. Data for different metabolites is stored in different workbooks, which are named with the metabolite name. The workbook shown here demonstrates isotopomer data for lactate. Column B shows the different fitted isotopomers, column C the respective percentages. Column D depicts multiplet components with the experimentally determined percentages shown in column E. Column F plots back-calculated multiplet percentages based in the fitted isotopomers. Columns G & H show experimental and simulated GC-MS data.

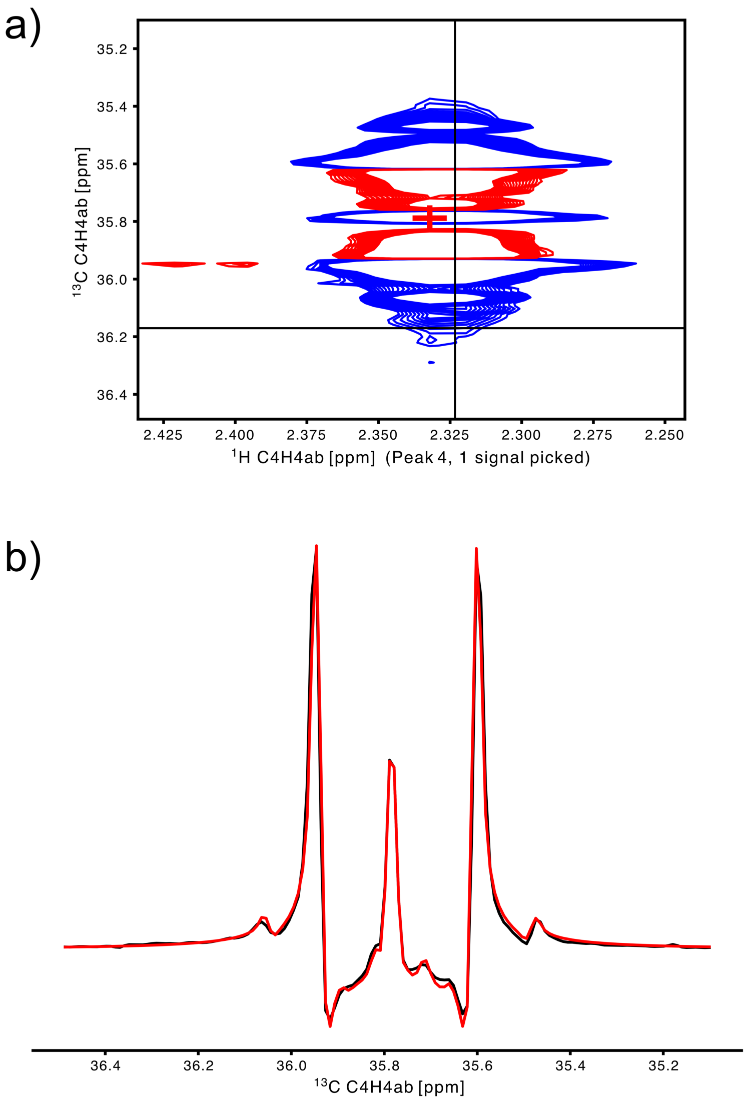

Figure S10. HSQC Spectrum plots. These two plots are the result of using the plot editor pane to select options for plotting output. The print command (Strg + P on Windows and Linux systems, ⌘ +P on macOS) creates pdf files.

Structure of mlInfo files

```
Name                    : L-Lactic Acid
AltName                 : lactate
Source                  : HMDB
IUPAC                   : (2S)-2-hydroxypropanoic acid
InChI_Identifier        : InChI=1S/C3H6O3/c1-2(4)3(5)6/h2,4H,1H3,(H,5,6)/t2-
/m0/s1
InChI_Key               : JVTAAEKCZFNVCJ-REOHCLBHSA-N
SMILES                  : C[C@H](O)C(O)=O
Formula                 : C3H6O3
Mass                    : 90.0779
pKa                     : 3.86
HMDB                    : HMDB00190
SMPDB                   : SMP00060 SMP00128
KEGG                    : map00010 map00620
CHEBI                   : 422
CID                     : 107689
C13Index                : 0;1;001 0;1;002 0;1;003
C13HSQC                 : 0  1  1
CO_HSQC                 : 1  2  2
C13Intensities          : 0           1      8.3349
H1Index                 : 2;1;000 3;3;123
N15Index                :
C13chemicalShift        : 180.0       71.2399       22.8972
H1chemicalShift         : 4.1011       1.314
N15chemicalShift        :
jCC                     : 1;2;54.1099 2;3;37.1594
jCH                     :
jCN                     :
jHH                     :
C13Offset               :
```

TopSpin Python script

```
# performs processing and automatic phase correction for multiple datasets
# requests first, last expno and increment from dialog
import os
import time

curdata = CURDATA()
if curdata == None:
    MSG("Please open a 1D NMR dataset!")
    EXIT()

cur_exp = int(curdata[1])
last_digit = cur_exp % 10
multiplier = 10
result = INPUT_DIALOG("", "Please enter first and last expnos as well as
increment:", ["first = ", "last = ", "increment = "], [curdata[1],
str(96*multiplier + last_digit), str(multiplier)])

if result == None:
    EXIT()

t     = time.time()
first = int(result[0])
last  = int(result[1])
incr  = int(result[2])


expno = first
n_exps = int((last - first) / incr + 1)
for i in range(n_exps):
    curdata[1] = str(expno)
    new_path = os.path.join(curdata[3], curdata[0], curdata[1])
    if os.path.isdir(new_path):
        if os.path.isfile(os.path.join(new_path, "fid")):
            RE(curdata)
            EFP()
            #XCMD("apk0.noe")
            #XCMD("fp")


    expno += incr


t2 = time.time()
mins = int((t2-t)/60.0)
output_str = "Elapsed time: " + str(t2 - t) + "s, " + str(mins) + " min, "
+ str(t2 - t - mins*60) + " sec"
MSG(message=output_str, title='Elapsed time')
```

MetaboLab Script

```
% Processing script for MetaboLab
% Comments start with a percentage sign
% Everything between START and END MLScript is executed
% everything outside is ignored
% The script starts in the next line
START MLScript

% Global Parameters
globalParameters
    graphicsDisplay: 1
endGlobalParameters

% Clear memory
clear

% set automation parameters
% Defaults are:
%      1. Don't overwrite an existing data set
%      2. Automatically increase exp numbers with every new spectrum
%      3. Don't process spectra automatically
autopars
    overWrite:   0
    autoRead:    1
    autoProcess: 0
endAutopars

% Read in 1D raw NMR data (Bruker/Agilent)
read1d
    dataPath: interactive
    dataSets: 1:100
    spcSet:   1
endRead1d

% Set processing parameters
procpars1d
    dataSetsP: all               % set parameters for all data sets
    % All processing parameters below are the default values used, when no
parameters are specified
    % Apodisation functions
         wdwf: EM                % window function
(EM/GM/SINE/QSINE/SEM/NONE)
           lb: 0.3               % line broadening
EM GM           SEM
           gb: 0.1               % gaussian broadening
GM
          ssb: 90                % sine bell shift
SINE QSINE SEM
       wdwsiz: 0                 % window size (0 for all data points)
SINE QSINE SEM
     wdw_nndp: -1                % treatment of non-negative data points (-
1/0/1)                        EM GM SINE QSINE SEM
    % Post-acquisition water suppression
          smo: 2                 % water suppression (0: none / 1: poly /
2: conv / 3: wavewat / 4: none)
    smo_order: 7                 % polynomial order for POLY water
suppression                           POLY    (smo = 1)
          sol: 32 32 0           % parameters for sol
CONV    (smo = 2)
```

```
           ww: 2 7 4 20 1        % parameters for wavewat
WAVEWAT (smo = 3)
    % Phase correction
    autophase: 0                % automatic phase correction (0: none / 1:
interactive / 4: apk4 / 5: apk5)
          ph0: 0                % zero order phase correction
          ph1: 0                % first order phase correction
    % Misc processing parameters
          dim: 1                % 1D spectra
        gibbs: 1                % half first data point of FID
           zf: 131072           % zero filling
          ref: auto             % referencing information (use DSS/TMSP if
'auto' / use middle of spectrum as water shift if 'water')
                                % provide referencing information as
ref_shift ref_point # points spectral width spectrometer frequency
                                % e.g.: 4.7458 65536 131072 7288.6 600.13
           bc: 32               % subtract last ... data points from FID
       ax_typ: 1                % plot spectra using ppm axis
         plot: 0                % plot current spectrum (0) or all spectra
(1)
 autoBaseline: 0                % Automatic baseline correction (requires
large spectral width)
endProcpars1d

% Process 1D spectra
proc1d
    dataSets: all               % Process all data sets
          ft: 1                 % Fourier transform data sets (including
phase corrections is phase: 1)
       phase: 1                 % Phase correct NMR spectra (including
automatic phase correction)
    baseline: 1                 % Automatic baseline correction (for
manual baseline correction, see pre-processing script)
   deleteSer: 0                 % Delete SER/FID file after processing (to
save computer memory)
endProc1d

% Save processed data to mat file
%save
    dataPath: path-to-data-file
    dataFile: metabolab1d.mat
endSave

% Display processed spectrum
disp
    currentSet: 1
    currentExp: 1
endDisp

% End of script
END MLScript
```

MetaboLabPy Script for *Daphnia* Spectra

```
kz = self.clear(True)
data_path = '/Users/ludwigc/Dropbox/mpy/daphnia'            # select
directory containing bruker data, interactive for file dialog
data_sets = [1, 2, 3, 4]                                              #
add comma separated list of experiment numbers (e.g. [1, 2, 3])
self.read_spcs([data_path],data_sets)                       # reading
Bruker spectra
msg = self.nd.set_zero_fill([131072])                       # zero fill to
131072 data points
msg = self.nd.set_lb([0.3])                                 # 0.3 Hz line
broadening
msg = self.nd.set_window_type(['Exponential'])              # window
function ('None', 'Exponential', 'Gaussian', 'Sine', 'QSine', 'SEM')
msg = self.nd.set_water_suppression('None')                       # set
water suppression ('None', 'Conv', 'Poly', 'WaveWat')
msg = self.nd.set_autobaseline(False)                       # perform
automatic baseline correction (False, True)
msg = self.nd.ft_all()                                      # Fourier
Transform all NMR spectra
msg = self.nd.auto_ref_all()                                # automatically
reference to TMSP
self.nd.e = 0                                               # make first
spectrum the current experiment
self.set_ph_ref_exp(1)                                      # select
reference spectrum for manual phase correction
self.plot_spc(keep_zoom=kz)
```

MetaboLabPy Example Script to read in NMRPipe Spectra

```
kz = self.clear(True)
data_path = 'Replace with directory containing Bruker and NMRPipe processed
data' # select directory containing bruker data, interactive for file
dialog
data_sets = []                                                        #
add comma separated list of experiment numbers (e.g. [1, 2, 3])
proc_data_name = 'test.dat'                                           #
name of NMRPipe processed data file (resides in e.g. 1.proc)
self.read_nmrpipe_spcs([data_path],data_sets, proc_data_name)
      # reading Bruker spectra
lds = len(self.nd.nmrdat[0])
for k in range(lds):                                                  #
    self.nd.nmrdat[0][k].display.n_levels = 34                        #
setting number of contour levels
    self.nd.nmrdat[0][k].display.min_level = 0.01
      # setting minimum contour level
    self.nd.nmrdat[0][k].display.max_level = 0.08
      # setting maximum contour level

self.nd.s = 0                                                         #
make first data set current data set
self.nd.e = 0                                                         #
make first experiment current experiment
self.plot_spc(keep_zoom=kz)
```

Table S1 – Isotopomer distributions from MetaboLab analysis and MetaboLabPy deep-learning algorithm analysis

Isotopomer	MetaboLab analysis [%]	Deep-learning algorithm analysis [%]
0 0 0	74.293	74.555
1 0 0	Not fitted	0.727
0 1 0	Not fitted	0.075
0 0 1	3.469	3.485
1 1 0	Not fitted	0.001
1 0 1	Not fitted	0.269
0 1 1	22.238	20.887
1 1 1	Not fitted	0.001

References

[1] Q. Bao *et al.*, ‘A robust automatic phase correction method for signal dense spectra’, *Journal of Magnetic Resonance*, vol. 234, pp. 82–89, Sep. 2013, doi: 10.1016/j.jmr.2013.06.012.
